# Supplementary material for: Pathogenic spectrum of blood stream infections and resistance pattern in Gram-negative bacteria from Aljouf region of Saudi Arabia
Source: PLoS One. 2020 Jun 9;15(6):e0233704. doi: 10.1371/journal.pone.0233704 (PMC7282660; doi:10.1371/journal.pone.0233704)
Supplement: Attachment — Submitted filename: Response to reviewer.docx [file pone.0233704.s001.docx]

**Response to reviewer**

| S/N Reviewer | Comments | Response |
| --- | --- | --- |
| Reviewer-1 | 1. If it is possible please specify the region (country) in the title of manuscript | The region and country has been specified in the title of manuscript. |
|  | 2. line 78-79: In the sentence „For Klebsiella pneumoniae, this rate is 65%, and the reported mortality caused by these organisms remains high” please indicate percentage of mortality rate | The statement has been modified and now includes the percentage of mortality rate. |
|  | 3. Table 2, sections Age and Microorganisms: please sort data from the highest to lowest as in the other sections | In the section the suggested changes have been made. |
|  | 4. Table 4: Whenever it’s possible I suggest to avoid commercial names of antibiotics such (i.e. Augmentin) and focus on active substances: amoxicillin and clavulanate potassium etc | The commercial name of antibiotic Augmentin is changed to the active substance |
|  | 5. Table 4, section Tygecycline: According to data shown in the table there was 3 resistant cases which is 17,6% of all cases, and there was no sensitive strains at all (0 –in the table). Where is 82,4% cases? Could you explain that? | I thank the reviewer for this important query. This comment was immensely beneficial. The correction has been made and a statement included in the results section. |
| Reviewer-2 | 1. The use of abbreviations in the abstract should be corrected. | All abbreviations have been removed from abstract |
|  | 2. The data can be explored further by applying non-parametric statistics such as Chi square especially in Table 2 (where applicable). That way the difference between the groups can be better appreciated instead of the percentages. In such cases, provide the p-values deduced as well. | Thanks for these comments. We wish to keep this table as it is because applying chi-square test does not have any impact on the understanding of the findings. Besides we wish to keep it as simple descriptive as possible. |
|  | 3. There are a lot of result repetitions in the discussion. Also, the discussions are un-conclusive; stating that the values are higher or lower than previous studies alone does not suffice, reasons should be given e.g is it due to variations in sample size, evaluation methods as so on. The entire discussion should be re-written based on this. | In its present form, every section of the discussion presents the major findings of the paper followed by comparison and reasoning in case of any observed variation in findings.  Based on the suggestion the repetition of the results in this section have been reduced and more focus has been paid to reasoning |
